# Covid-19: Invisible, Elusive and the Advancing Enemy

**DOI:** 10.12669/pjms.36.COVID19-S4.2758

**Published:** 2020-05

**Authors:** Khalid Mahmood Shafi, Anusha Sultan Meo, Rohaan Khalid

**Affiliations:** 1 Khalid Mahmood Shafi, National Defence University, Islamabad - Pakistan; 2 Anusha Sultan Meo, Army Medical College Rawalpindi, Pakistan; 3 Rohaan Khalid, Aitchison College, Lahore - Pakistan

Since Dec 2019, novel coronavirus (Covid-19) involved 207 countries and territories, infected 976249 people with a mortality rate of 50489 (5.17%).^1^ The Covid-19 created health, economic, political, and social challenges worldwide.

The Covid-19 pandemic revolutionized the threat dimensions. The policy makers and strategists had been debating traditional and non-traditional security threats, especially in the last five decades, wherein the wideners have over taken the traditionalists.^2^ Traditional security threats revolved around violation of human rights through the act of terrorism, nuclear war, civil war, inter or intra-state wars, struggle for power maximization and other state-centric threats. However, the non-traditional security threats encompassed poverty, hunger, epidemics, food security, climate change impacts and other assorted challenges which go beyond the serious consideration of the international community including leading global institutions like the United Nations and others.

On the contrary, the greatest threat for the maintenance of global peace and security for human survival has emerged from this pandemic of corona virus, for which neither individuals nor nations were prepared. The causes and effects of this real threat have surpassed the heralds of nuclear war, war on terror and even the climate change.

China was the first to thwart and contain, whereas Italy and Iran were later in the run; they had prior warnings but failed to restrict the virus. The basic reason other than the evident medical facilities and economic resources were the conceptual policy making and implementation. China being a strict state implemented measures aggressively and effectively. The military worked under the government directives and results were obtained. In Iran, the disconnection between military and government officials was evident. In Iran the advice of military to restrict movement went unheeded and later on it was a disjointed and half-hearted effort.^2^ Italy on the other hand was a liberal democracy, which when tried to implement the isolation formula was defeated due to civic rights usurping slogans and a care free attitude, until it was too less and too late.

Lessons were derived from Word War I (1914-18) and World War II (1939-45), but what were the lessons from the threat which affected quarter of the world’s population and death of about 50 million in 1919. Unfortunately, not much was derived from the Spanish flu, rather it was forgotten in the annals of history.

Now, what are lessons a miniscule, emerging pathogen has taught to 7.8 billion humans at the start of new decade after all? Threats to humanity are not confined, and limited by borders and financial situations. This crisis motivates us to see through the fog of fake individualism at national and international level. Role of institutions have been rearranged the United Nations Security Council (UNSC) has been replaced by World Health Organization (WHO), and medical fraternity has gained ancillary importance in collaboration with military. Medical and pra-medical workers are the new frontline soldiers. Netizens are the mouths and ears, which with medical authentication can become credible and effective. In dealing with any threat, we direly need to have singleness of conception, incorporating medical experts and harmonious execution. The fear-provoking pathogens such as Covid-19 can be seen as a revelation, as some time crisis turning the challenges into opportunities.
